# Eligibility for ^177^Lu-PSMA Therapy Depends on the Choice of Companion Diagnostic Tracer: A Comparison of ^68^Ga-PSMA-11 and ^99m^Tc-MIP-1404 in Metastatic Castration-Resistant Prostate Cancer

**DOI:** 10.2967/jnumed.122.264296

**Published:** 2023-02

**Authors:** Gary J.R. Cook, Wai-Lup Wong, Bal Sanghera, Stephen Mangar, Amarnath Challapalli, Amit Bahl, Paul Bassett, Darren Leaning, Christian Schmidkonz

**Affiliations:** 1 Cancer Imaging Department, School of Biomedical Engineering and Imaging Sciences, King’s College London, London, United Kingdom;; 2 Paul Strickland Scanner Centre, Mount Vernon Hospital, Northwood, Northwood, United Kingdom;; 3 Department of Oncology, Charing Cross Hospital, Imperial College Healthcare NHS Trust, London, United Kingdom;; 4 Department of Clinical Oncology, Bristol Cancer Institute, Bristol, United Kingdom;; 5 Statsconsultancy Ltd, Amersham, United Kingdom;; 6 Department of Clinical Oncology, James Cook University Hospital, South Tees NHS Trust, Middlesbrough, United Kingdom;; 7 Department of Nuclear Medicine, University Hospital Erlangen, Erlangen, Germany; and; 8 Department of Industrial Engineering and Health, Technical University of Applied Sciences Amberg-Weiden, Weiden, Germany

**Keywords:** ^177^Lu-PSMA-617, ^68^Ga-PSMA-11, ^99m^Tc-MIP-1404

## Abstract

^177^Lu-prostate-specific membrane antigen-617 (^177^Lu-PSMA-617) is an effective therapy for metastatic castration-resistant prostate cancer (mCRPC), with evidence of improved survival over standard care. The VISION trial inclusion criteria required a metastatic lesion-to-liver ratio of greater than 1 on ^68^Ga-PSMA-11 PET scans. We aimed to determine whether an equivalent ratio is suitable for a SPECT tracer, ^99m^Tc-MIP-1404, and to compare lesion and lesion–to–normal-organ ratios between the 2 radiotracers. **Methods:** Two cohorts of patients with mCRPC matched for age, prostate-specific antigen level, and total Gleason score, with either ^99m^Tc-MIP-1404 SPECT/CT (*n* = 25) or ^68^Ga-PSMA-11 PET/CT (*n* = 25) scans, were included for analysis. Up to 3 lesions in each site (prostate/prostate bed, lymph nodes, bone and soft-tissue metastases) as well as normal liver, parotid gland, spleen, and mediastinal blood-pool SUV_max_ were measured. **Results:** ^99m^Tc-MIP-1404 SPECT lesion SUV_max_ was not significantly different from ^68^Ga-PSMA-11 PET (median, 18.2 vs. 17.3; *P* = 0.93). However, ^99m^Tc-MIP-1404 liver SUV_max_ was higher (median, 8.5 vs. 5.8; *P* = 0.002) and lesion-to-liver ratios were lower (median, 2.7 vs. 3.5; *P* = 0.009). There was no significant difference in parotid gland or splenic SUV_max_ or lesion–to–parotid gland ratios between the 2 tracers although there was a small difference in lesion-to-spleen ratios (*P* = 0.034). **Conclusion:** There are differences in biodistribution and, in particular, liver activity, between ^68^Ga-PSMA-11 and ^99m^Tc-MIP-1404. Therefore, if ^99m^Tc-MIP-1404 is used to assess eligibility for ^177^Lu-PSMA-617 therapy, a lower adjusted lesion-to-liver ratio should be used.

## Visual Abstract

**Figure:**
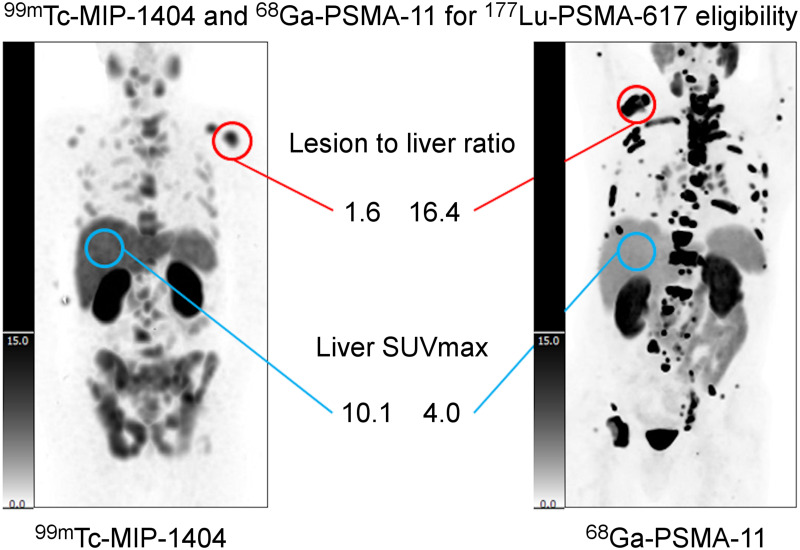

The therapeutic options for metastatic castration resistant prostate cancer (mCRPC) are rapidly expanding, especially in the area of targeted radionuclide therapy. In particular, exploiting prostate-specific membrane antigen (PSMA) overexpression in metastatic disease is an appealing option for targeted therapy (1). The recently reported VISION trial confirmed an improvement in progression-free survival and overall survival after treatment with radiolabeled PSMA therapy, ^177^Lu-PSMA-617 (2). In keeping with optimal practice and the principles of theranostics, and given the knowledge that some prostate cancers do not express PSMA, target expression was mandated by ^68^Ga-PSMA-11 PET imaging as an inclusion criterion (3). Specifically, the trial required at least 1 PSMA-positive metastasis, defined as uptake greater than liver with no PSMA-negative lesion (uptake ≤ liver) in any measurable metastasis (lymph node > 2.5 cm, solid organ > 1.0 cm, bone > 1.0 cm soft-tissue component). In the trial, 126 of 995 subjects did not meet imaging criteria.

The inclusion criteria were based on pragmatic reasons, including the widespread use and availability of ^68^Ga-PSMA-11 (2*,*^3^) and that screen failures were subsequently shown to have worse outcomes (4).

As eligibility for ^177^Lu-PSMA-617 therapy requires only a binary decision depending on the level of lesion uptake rather than requiring maximal sensitivity for lesion detection, several alternative PET and SPECT PSMA ligands exist that could potentially be used to confirm metastatic PSMA expression, some of which may be less costly or more readily available and accessible in different geographic areas of the world. These include other ^68^Ga-, ^18^F-, and ^99m^Tc-labeled ligands that have demonstrated utility in detection of PSMA-expressing metastases in prostate cancer (5–12). For example, ^99m^Tc-MIP-1404 has shown efficacy in patients with intermediate- and high-risk prostate cancer undergoing prostatectomy and extended pelvic lymph node dissection (5) and has favorable radiation dosimetry (0.0088 mSv/MBq) compared with ^68^Ga-PSMA-11 (0.022 mSv/MBq) (13*,*^14^). However, these ligands have biodistributions different from ^68^Ga-PSMA-11, and particularly in those where biliary rather than renal excretion predominates, the use of a lesion- to-liver ratio of greater than 1 may deny patients access to ^177^Lu-PSMA-617 treatment when ^68^Ga-PSMA-11 would have confirmed eligibility. There is therefore a need to determine appropriate quantitative criteria for different PSMA ligands to ensure equity of eligibility to ^177^Lu-PSMA-617 therapy.

Our aim was to compare the biodistributions of ^68^Ga-PSMA-11 and ^99m^Tc-MIP-1404 (ROTOP Pharmaka GmbH) in metastases and potential reference organs in matched cohorts of patients with mCRPC. Our hypothesis was that differences in biodistributions would be present, requiring an appropriate equivalent metric for ^99m^Tc-MIP-1404 to define treatment eligibility.

## MATERIALS AND METHODS

Institutional approval was acquired for analysis of anonymized retrospective data without the need for further consent. Two cohorts of 25 consecutive patients who had undergone ^68^Ga-PSMA-11 PET/CT or ^99m^Tc-MIP-1404 SPECT/CT scans were included in the analysis. Inclusion criteria were CRPC with metastatic disease being considered for systemic therapy including ^177^Lu-PSMA-617. Patients were excluded if they did not have mCRPC or information on a prostate-specific antigen (PSA) level within 1 mo of the PSMA scan or no history of the original Gleason score. Age, PSA level closest to the time of scanning, and original Gleason score data were collected.

### ^68^Ga-PSMA-11 PET/CT Scan

No specific patient preparation was required except bladder voiding immediately before imaging. All patients were injected intravenously with ^68^Ga-PSMA-11 (mean, 169.6 ± 16.5 MBq). At 60 min, a scan was acquired from pelvis to skull base at 4 min per bed position with an axial field of view of 15.7 cm and an 11-slice overlap between bed positions, using a Discovery 710 PET/CT scanner (GE Healthcare). A low-dose CT scan (140 kV; mAs, 15–100; noise index, 40; rotation time, 0.5 s; and collimation, 40 mm) was obtained at the start of imaging to provide attenuation correction and an anatomic reference. PET image reconstruction used a Bayesian penalized likelihood algorithm (Q.CLEAR; GE Healthcare) with a β penalization factor of 800 as previously described (15*,*^16^).

### ^99^Tc-MIP-1404 SPECT/CT Scan

No specific patient preparation was required except bladder voiding immediately before imaging. All patients were injected intravenously with ^99m^Tc-MIP-1404 (mean 705 ± 61 MBq). At 2–4 h, SPECT scans were acquired on a Symbia T2 SPECT/CT system (Siemens Healthcare) from midthigh to skull vertex with low-energy high-resolution collimation, a 128 × 128 matrix with 4.8-mm pixel size, and 120 projections over 360° for 15 s per projection. SPECT scans were followed by low-dose CT (130 kV, 30 mAs) using adaptive dose modulation (CAREDose 4D; Siemens Healthcare). CT data were reconstructed with 3- and 5-mm slice thicknesses using B70s and B41s kernels for image analysis. The SPECT dataset was reconstructed using an ordered-subset expectation maximization algorithm with 4 subsets and 8 iterations, including point-spread-function modeling with CT-based attenuation correction and dual-energy window scatter correction as previously described (10). Both PET and SPECT scanners underwent routine quality control measures.

### Scan Analysis

For each subject, SUV_max_ was measured in any malignant lesion in the prostate or prostate bed and up to 3 lesions in each of pelvic nodes (N1), extrapelvic nodes (M1a), and skeletal (M1b) and visceral metastases (M1c) using Hermes Gold software (Hermes Medical Solution). A semiquantitative expression score was measured by comparison to normal tissues including the mediastinal blood pool (MBP), liver, spleen, and parotid glands, according to the PROMISE proposed classification for PSMA ligand PET/CT interpretation (17). For the liver, a 3-cm spheric volume of interest was placed in the center of the right lobe avoiding metastases if present, with 2-cm volumes of interest used for spleen, MBP (aortic arch), and parotid glands. SUV_max_ was calculated for each. For each malignant lesion, a lesion-to-liver, lesion-to-spleen, lesion–to–blood-pool, and lesion–to–parotid gland ratio was calculated.

### Statistical Analysis

Statistical analyses were performed using SPSS (version 27; IBM Corp). Data were tested for normality, and comparisons made with either the *t* test or the Mann–Whitney test and values presented as mean ± SD or median and range accordingly. A *P* value of < 0.05 was taken for statistical significance.

## RESULTS

The ^99m^Tc-MIP-1404 group and the ^68^Ga-PSMA-11 group of mCRPC patients were matched for age (median, 72 and 71 y, respectively), PSA level (mean, 413.4 and 415.6 ng/mL, respectively), and Gleason score (median, 9 and 9, respectively). The previous rates of androgen deprivation therapy and other local or systemic therapies (except prostatectomy rate) were also similar between the 2 groups (Table 1). The 2 groups showed a similar distribution of metastatic disease on either ^99m^Tc-MIP-1404 SPECT or ^68^Ga-PSMA-11 PET in the prostate/prostate bed (7 and 10 patients, respectively), N1 (8 and 13 patients, respectively), M1a (8 and 9 patients, respectively), M1b (23 and 23 patients, respectively), and M1c (4 and 4 patients, respectively).

**TABLE 1.** Characteristics in ^99m^Tc-MIP-1404 and ^68^Ga-PSMA-11 Groups

Characteristic	^99m^Tc-MIP-1404	^68^Ga-PSMA-11
Median age (y)	72 (range, 62–91)	71 (range, 54–84)
Median Gleason score	9 (SD, 8–9)	9 (SD, 7–9)
Mean PSA	413.6 (SD, 581.6)	415.6 (SD, 1,101.7)
Prior ADT	24	25
Current ADT	6	8
Prior prostatectomy	14	4
Prior prostate radiotherapy	13	13
Prior chemotherapy	20	15
miTNM		
Prostate	7	10
N1	8	13
M1a	8	8
M1b	23	23
M1c	3	4

ADT = androgen deprivation therapy; miTNM = molecular imaging tumor, node and metastasis staging.

**FIGURE 1.**
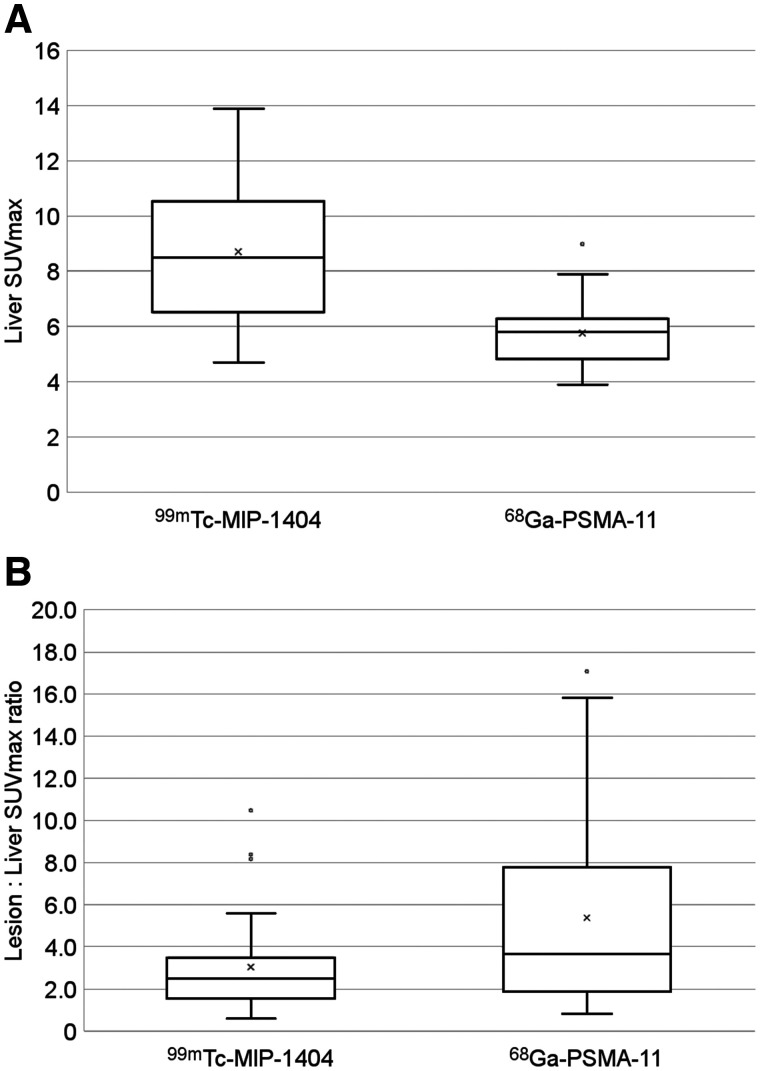
Box plots of ^99m^Tc-MIP-1404 and ^68^Ga-PSMA-11 liver SUV_max_ (*P* = 0.002) (A) and lesion-to-liver SUV_max_ ratios (*P* = 0.009) (B) in patients with mCRPC. median = central line; mean = X; box = interquartile range (IQR); whiskers = maximum and minimum values; ^O^ = outliers > 1.5 IQR.

Lesion SUV_max_ measurements were not statistically different between the 2 groups (median, 18.2, range, 4.1–52.1 for the ^99m^Tc-MIP-1404 group; and median, 17.3, range, 3.4–128 for the ^68^Ga-PSMA-11 group; *P* = 0.93). However, liver SUV_max_ was higher in the ^99m^Tc-MIP-1404 scans (median, 8.5, range, 4.7–13.9 for the ^99m^Tc-MIP-1404 group; and median, 5.8, range, 3.9–9.0 for the ^68^Ga-PSMA-11 group; *P* = 0.002) (Fig. 1A). Lesion-to-liver ratios were lower in the ^99m^Tc-MIP-1404 group (median 2.5, range 0.6–10.5 for the ^99m^Tc-MIP-1404 group; and median, 3.7, range, 0.8–17.1 for the ^68^Ga-PSMA-11 group; *P* = 0.009) (Fig. 1B). Lesion-to-liver ratios of ^99m^Tc-MIP-1404 SPECT were on average 57% of those with ^68^Ga-PSMA-11 PET.

There was no significant difference in parotid gland SUV_max_ between the ^99m^Tc-MIP-1404 and the ^68^Ga-PSMA-11 group (mean, 13.4 ± 7.1 and 16.8 ± 6.4, respectively; *P* = 0.23) or lesion–to–parotid gland ratios (median, 1.8, range; 0.3–6.4; and median, 1.4, range, 0.3–7.0, respectively; *P* = 0.5). Similarly, there was no difference between spleen SUV_max_ (median, 11.5, range, 5.4–22.5; and median, 9.3, range, 4.9–16.0, respectively; *P* = 0.072), but there was a small difference in lesion-to-spleen ratios (median, 1.4, range, 0.3–6.1; and median, 2.0, range, 0.4–14.9, respectively; *P* = 0.034). There was a difference between MBP SUV_max_ measurements (mean, 1.1 ± 0.3 and 1.6 ± 0.3, respectively; *P* = 0.003) and lesion- to-MBP ratios (median, 19.9, range, 3.8–114.5; and median, 13.5, range, 2.7–57.6, respectively; *P* = 0.011) (Table 2).

**TABLE 2.** Lesion, Normal-Organ (Liver, Spleen, Parotid Gland, MBP), and Lesion–to–Normal-Organ Ratios Between ^99m^Tc-MIP-1404 and ^68^Ga-PSMA-11 mCRPC Patients

	^99m^Tc-MIP-1404		^68^Ga-PSMA-11		
Parameter	Median	Mean ± SD	Median	Mean ± SD	*P*
Lesion SUV_max_	18.2 (4.1–52.1)		17.3 (3.4–128.0)		0.93
Liver SUV_max_	8.5 (4.7–13.9)		5.8 (3.9–9.0)		0.002
Lesion-to-liver SUV_max_ ratio	2.5 (0.6–10.0)		3.7 (0.8–17.1)		0.009
Parotid gland SUV_max_		13.4 ± 7.1		16.8 ± 6.4	0.23
Lesion–to–parotid gland SUV_max_ ratio	1.8 (0.3–6.4)		1.4 (0.3–7.0)		0.5
Spleen SUV_max_	11.5 (5.4–22.5)		9.3 (4.9–16.0)		0.072
Lesion-to-spleen SUV_max_ ratio	1.4 (0.3–6.1)		2.0 (0.4–14.9)		0.034
MBP SUV_max_		1.1 ± 0.3		1.6 ± 0.3	0.003
Lesion-to-MBP SUV_max_ ratio	19.9 (3.8–114.5)		13.5 (2.7–57.6)		0.011

Data in parentheses are ranges.

## DISCUSSION

Although we have not measured a difference in lesion avidity between ^99m^Tc-MIP-1404 SPECT and ^68^Ga-PSMA-11 PET PSMA ligands in patients with mCRPC, there are differences in biodistribution. In particular, liver activity and hence lesion-to-liver ratios differ between the 2 tracers with higher liver uptake but lower lesion-to-liver ratios with ^99m^Tc-MIP-1404 SPECT. This is potentially of importance as the eligibility for ^177^Lu-PSMA-617 therapy in the VISION trial depended on a lesion-to-liver ratio greater than 1 using ^68^Ga-PSMA-11 (2*,*^3^). Another phase 2 study used a lesion-to-liver ratio of 1.5 and absence of ^18^F-FDG–positive and ^68^Ga-PSMA-11–negative lesions to determine eligibility (18).

Different eligibility criteria would therefore need to be considered for different PSMA ligands so that patients are not deemed ineligible from treatment when they may benefit if confirmation of PSMA expression at all metastatic sites is mandated by reimbursement guidelines before treatment.

Although the predominant renal excretion route of ^99m^Tc-MIP-1404 is not dissimilar to that of ^68^Ga-PSMA-11 (10), there appear to be sufficient differences to provide variation in lesion-to-liver ratios between the 2 tracers. This is probably even more relevant for other PSMA ligands that have a predominant hepatobiliary route of excretion such as ^18^F-PSMA-1007, for which liver SUV_max_ has been shown to be more than double that of ^68^Ga-PSMA-11 in a comparative study (8). We found the mean ^99m^Tc-MIP-1404 lesion-to-liver ratio to be 57% of that of ^68^Ga-PSMA-11, suggesting that a correspondingly lower lesion-to-liver ratio would be required to achieve equitable eligibility for ^177^Lu-PSMA-617 therapy if applying the VISION trial, or other previously reported, eligibility criteria (2*,*^18^). Although an optimal level of lesion uptake has not been defined for ^177^Lu-PSMA-617 efficacy, it is generally accepted that a theranostic pair is required to ensure targetable disease and to avoid futile therapy in the minority of patients with non–PSMA-expressing disease (4*,*^11^).

To standardize PSMA ligand PET reporting, it has been suggested that uptake in metastatic lesions should be graded by comparison to blood pool, liver, or parotid gland (or spleen in liver-dominant excretion ligands) (17). We have therefore measured uptake in these organs in our series. Our results compare closely with another published series of ^68^Ga-PSMA-11 with respect to liver, spleen, and parotid gland SUV_max_ measurements with a small difference in blood-pool values, possibly due to methodologic differences in measurement of the latter (6). Interestingly, the ^99m^Tc-MIP-1404 ligand shows no significant difference from ^68^Ga-PSMA-11 with respect to lesion uptake, parotid gland uptake, lesion–to–parotid gland ratio, spleen, and lesion-to-MBP ratios. Apart from the previously mentioned lesion-to-liver ratio differences, lesion-to-spleen ratios and MBP SUV_max_ were lower for ^99m^Tc-MIP-1404, reflecting differences in biodistribution and pharmacokinetics of the 2 PSMA ligands. We also noted higher variability in splenic and parotid gland uptake between patients for both tracers compared with liver SUV_max_, suggesting these organs may be less suitable than the liver as semiquantitative comparators. Rather than using a liver threshold, other studies have used a lesion SUV_max_ of 20 as an eligibility criterion (19*,*^20^), and it is possible that this would present an effective alternative treatment threshold. Indeed, our data show a similar SUV_max_ between ^99m^Tc-MIP-1404 and ^68^Ga-PSMA-11 (Table 2), but further work would be required to determine comparable lesion avidity in other PSMA tracers.

Our analysis has some limitations. First, the 2 types of PSMA scan were not performed in the same patients. Nevertheless, both the groups analyzed included patients with mCRPC who would potentially be eligible for ^177^Lu-PSMA-617 therapy and were reasonably well matched for age, PSA level, Gleason score, and other relevant characteristics. However, perfect matching is unlikely and a study of both scans in the same patients, or a study evaluating ^68^Ga-PSMA-11 uptake in ^99m^Tc-MIP-1404–negative patients, would provide more robust data. Second, not all of the patients in our cohorts received ^177^Lu-PSMA-617 therapy and so we do not know if potential differences in eligibility for therapy would have had an impact on clinical outcomes. In addition, we cannot exclude systematic differences in SUV calculation between the SPECT and PET modalities, weakening a comparison of SUV_max_ between lesions and organs in the 2 cohorts. However, the ratios that we measured would not be significantly affected by systematic differences.

## CONCLUSION

There are differences in biodistribution between ^99m^Tc-MIP-1404 and ^68^Ga-PSMA-11 in patients with mCRPC who might be eligible for ^177^Lu-PSMA-617 therapy such that different semiquantitative criteria may need to be adopted for different ligands if lesion-to-liver ratios are the main parameter under consideration. This is likely to be even more important for PSMA ligands that are predominantly excreted via the hepatobiliary route. Prospective analysis of the optimal imaging metrics to predict treatment outcomes will be an important goal in future trials, particularly if SPECT agents that offer increased availability and access with reduced costs are used.

## DISCLOSURE

Financial support was provided by the following: Cancer Research U.K. National Cancer Imaging Translational Accelerator (A27066), the Wellcome/Engineering and Physical Sciences Research Council Centre for Medical Engineering at King’s College London (WT 203148/Z/16/Z), and National Institute for Health Research Biomedical Research Centre at Guy’s & St Thomas’ Hospitals and King’s College London. No other potential conflict of interest relevant to this article was reported.

## Notes

KEY POINTS **QUESTION:** Can differences in PSMA imaging tracer biodistribution affect selection of patients for ^177^Lu-PSMA-617 therapy if lesion-to-liver ratios are used for treatment selection? **PERTINENT FINDINGS:** This comparison of retrospective matched ^68^Ga-PSMA-11 PET and ^99m^Tc-MIP-1404 SPECT data in 2 cohorts of 25 patients with mCRPC has shown differences in biodistribution that led to greater liver activity but lower lesion-to-liver ratios for ^99m^Tc-MIP-1404. **IMPLICATIONS FOR PATIENT CARE:** Different semiquantitative data may be required for different PSMA imaging tracers used as companion diagnostics to ensure equal eligibility for ^177^Lu-PSMA-617 therapy in mCRPC.
